# Salvage hepatectomy for local recurrence after particle therapy using proton and carbon ion beams for liver cancer

**DOI:** 10.1002/ags3.12468

**Published:** 2021-05-07

**Authors:** Motofumi Tanaka, Shohei Komatsu, Masahiro Kido, Hirochika Toyama, Masahiro Tominaga, Yoichiro Uchida, Kazuki Terashima, Yusuke Demizu, Tomoaki Okimoto, Takumi Fukumoto

**Affiliations:** ^1^ Division of Hepato‐Biliary‐Pancreatic Surgery Department of Surgery Kobe University Graduate School of Medicine Kobe Japan; ^2^ Department of Gastroenterological Surgery Hyogo Cancer Center Akashi Japan; ^3^ Department of Gastroenterological Surgery and Oncology The Tazuke Kofukai Medical Research Institute Kitano Hospital Osaka Japan; ^4^ Department of Radiology Hyogo Ion Beam Medical Center Tatsuno Japan; ^5^ Department of Radiation Oncology Hyogo Ion Beam Medical Center Kobe Proton Center Kobe Japan

**Keywords:** carbon ion beam, hepatocellular carcinoma, particle therapy, proton beam, salvage hepatectomy

## Abstract

**Aim:**

With the increased use of particle therapy for liver cancer, local recurrence after particle therapy increased. Salvage hepatectomy is an acceptable treatment option for local recurrence following particle therapy; however, its safety and effectiveness remain unclear. Therefore, this multi‐center study aimed to verify the feasibility and efficacy of salvage hepatectomy and assess clinical issues associated with its application.

**Methods:**

We retrospectively assessed the perioperative outcomes, prognosis, and pathological characteristics of 15 patients who underwent salvage hepatectomy for local recurrence after particle therapy between 2006 and 2019.

**Results:**

Hepatocellular carcinoma and metastatic liver tumors were noted in eight and seven patients, respectively. The mean total dose and number of fractions were 66.5 Gy and 12, respectively, and the mean interval between particle therapy and surgery was 30.1 months. Major hepatectomy was performed in seven cases. Moreover, the mortality rate was 0%, and surgical complications of Clavien‐Dindo grade IIIa or higher were observed in four cases (27%)—two bile leakages, one pleural effusion, and one refractory skin fistula. The median overall survival time and 5‐year overall survival rate after salvage hepatectomy were 29.9 months and 43.1%, respectively. Histological examination of the irradiated liver tissue surrounding the tumor showed sinusoidal dilatation, loss of hepatocyte, and fibrosis in most cases.

**Conclusion:**

Salvage hepatectomy after particle therapy is a feasible therapy; however, the risk of refractory complications associated with particle therapy is relatively high. Therefore, the first‐line treatment for resectable liver cancer should be carefully determined considering second‐line treatment after local recurrence.

## Graphical abstract

This is the first study that verified feasibility and efficacy of salvage hepatectomy for local recurrence after particle therapy and indicated clinical issues and pathological features of this therapy. These data would be a guidepost for surgeons to treat such patients.

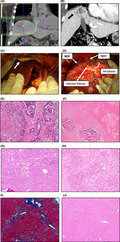

## 1 INTRODUCTION

Particle therapy (PT), that is, therapy employing a proton or carbon ion beam, displays superior depth‐dose distribution in specific ranges compared to photon radiotherapy. This characteristic, termed the Bragg peak, induces elevated energy for the ablation of tumors with specific dimensions and causes minimum damage to the surrounding normal tissue. PT has been widely used for malignant tumors originating in several organs, such as the prostate, lungs, bones, and liver.^1^, ^2^, ^3^

Among liver cancers, hepatocellular carcinoma (HCC) is a good candidate for PT with high local control rates of 71.4%‐94.8% at 2‐5 years^3^, ^4^, ^5^, ^6^ and reduced incidence of severe toxicity. PT has the advantage of preserving non‐cancerous liver parenchyma in patients with deteriorated function due to cirrhosis or pretreatment chemotherapy,^7^, ^8^ whereas, curative local therapies for HCC, including liver resection and radiofrequency ablation, are frequently limited due to liver dysfunction and tumor factor. The role of PT for HCC is presented in several clinical guidelines. The 2017 clinical practical guidelines for liver cancer published in Japan weakly recommended PT for cases of HCCs, which are not indicated for other types of locoregional therapies. However, in practice, PT is performed even for patients who can be treated with liver resection or radiofrequency ablation following preferred minimally invasive modalities. This type of treatment strategy increases the number of patients receiving PT for liver cancer.

Particle therapy for liver cancer is safe with low invasiveness and can achieve a relatively significant local control compared with hepatectomy. However, a standard therapy for local recurrence following PT has not been established. In patients with sufficient liver function, salvage hepatectomy remains theoretically a preferred curative‐intent treatment option. The efficacy and safety of salvage hepatectomy for patients with local recurrence after PT have not been fully discussed. Furthermore, case reports on salvage hepatectomy have been scarce.^9^, ^10^ In this study, we analyzed the feasibility and effectiveness of salvage hepatectomy and discussed clinical issues and pathological features of the liver following PT.

## 2 MATERIALS AND METHODS

### 2.1 Patients

A multi‐center retrospective study was conducted. Fifteen patients who underwent salvage hepatectomy for local recurrence after PT between January 2006 and June 2019 at Kobe University Hospital, Hyogo Cancer Center, and Kitano Hospital were enrolled. All patients, apart from one, had received PT in Hyogo Ion Beam Medical Center. At the time of treatment selection, some patients chose to receive PT as a less‐invasive therapy, even for resectable tumors. In this period, 1377 patients with HCC were treated at this center and 69 faced local recurrence (5%). Among them, nine patients underwent salvage hepatectomy and seven of them were recruited in this study. Regarding metastatic tumor, 176 patients were treated at Hyogo Ion Beam Medical Center and 41 of them had local recurrence (23%). Among them, eight patients underwent salvage hepatectomy and seven of them were recruited in this study. Two patients with HCC and one with liver metastasis who underwent salvage hepatectomy in other institutions were excluded from this study.

Clinical and pathological data were collected and analyzed. This study was approved by the Institutional Review Board of each institution.

### 2.2 Data collection

Clinical characteristics, such as age, sex, liver function, and tumor factor and type, were collected. Additionally, PT information, such as beam type, intensity, fraction, radiation planning, and interval time from irradiation to surgical operation, were also obtained. Moreover, postoperative outcomes, such as morbidity, mortality, survival status, and recurrence status, were obtained and analyzed.

### 2.3 Statistical analyses

The follow‐up time was calculated from the day of salvage hepatectomy. The probabilities of overall survival (OS) and recurrence‐free survival (RFS) were estimated using the Kaplan‐Meier method and compared using the log‐rank test. The correlation between the irradiation dose and the resection line and postoperative complications was estimated using the chi‐square (χ^2^) test. All statistical analyses were performed using the SPSS statistics software version 22.0 (IBM Corp., Armonk, NY, USA).

### 2.4 Pathological examination

Resected specimens were examined for pathological changes of the recurrent tumor and the adjacent non‐cancerous liver tissue by applying PT. Staining methods, such as hematoxylin and eosin (H&E) and Masson, were employed.

## 3 RESULTS

### 3.1 Clinical characteristics of the study participants

The details of patients who underwent salvage hepatectomy are provided in Table 1. The mean age of patients was 64.4 (range: 35‐79) years. The cancer types included eight HCCs and seven liver metastases, as well as one pancreatic ductal and six colorectal adenocarcinomas. The median size and number of primary tumors before PT were 3.5 (range: 1.0‐9.7) cm and 1.1 (range: 1‐2), respectively.

**TABLE 1:** Patient list (n = 15)

No.	Age	Sex	Primary tumor characteristics				Particle therapy				Interval (months)	Preoperative data		
			Cancer types	Location	Tumor size (cm)	Tumor number	Reason	Type	Intensity (Gy [RBE])	Fraction		Child‐Pugh score	Tumor size (cm)	Tumor number
1	76	M	HCC	S6	5.6	1	Unresectable	Proton	60	10	4.4	6	2.0	2
2	78	F	CRLM	S6/7	3.2	1	Patient's will	Carbon	60	8	9.7	5	5.2	1
3	35	F	HCC	S3	1.0	1	Unresectable	Proton	76	20	6.2	5	1.1	1
4	77	M	PDAC‐LM	S6	2.3	1	Patient's will	Carbon	60	8	7.4	5	5.0	1
5	51	F	HCC	S7/8	5.7	1	Patient's will	Proton	66	10	18.4	5	1.0	4
6	64	M	CRLM	S6/7/8	2.1	1	Patient's will	Carbon	64	8	26.9	5	4.5	1
7	66	M	CRLM	S8	3.2	2	Patient's will	Proton	66	10	25.1	5	5.4	1
8	54	M	CRLM	S6/7	5.2	1	Patient's will	Carbon	68	8	7.9	5	4.0	1
9	67	M	CRLM	S8/5	2.5	1	Patient's will	Carbon	72	8	12.3	5	7.2	5
10	67	M	HCC	S3	1.9	1	Patient's will	Carbon	76	20	101.5	5	1.0	1
11	63	M	CRLM	S3/4	1.6	1	Patient's will	Carbon	68	8	34.7	5	3.8	3
12	79	M	HCC	S2/3/4	9.7	1	Patient's will	Carbon	76	38	18.9	5	3.9	5
13	61	M	HCC	S6	3.4	1	Patient's will	Carbon	52.8	4	55.0	5	1.7	2
14	54	M	HCC	S3	4.0	1	Patient's will	Proton	66	10	10.5	5	3.6	1
15	74	M	HCC	S5	1.3	1	Patient's will	Carbon	66	10	113	5	1.2	1

Abbreviations: CRLM, colorectal liver metastasis; HCC, hepatocellular carcinoma; PDAC‐LM, pancreatic ductal adenocarcinoma liver metastasis; RBE, relative biologic effectiveness.

Particle therapy was conducted as the primary treatment for patients who wanted to undergo this procedure in 13 cases and in two cases of unresectable HCC. The former 13 patients had resectable tumors; however, they chose to undergo PT as a less invasive therapy. The latter two cases were of a patient with HCC with portal venous tumor thrombus and another with HCC with lung metastasis. Since the portal venous tumor thrombus was shrunk by RT and R0 resection could be performed for recurrent tumor, and lung metastasis was already resected after PT without recurrence, we decided to perform salvage hepatectomy. PT was performed with curative intent using protons or carbon ions. The mean total dose and number of fractions of PT were 66.5 Gy (relative biologic effectiveness, RBE; range, 52.8‐76) and 12 (range: 4‐38), respectively. Proton beam therapy of 60‐76 Gy (RBE) in 10‐20 fractions was performed for five patients and carbon ion radiotherapy of 52.8‐76 Gy (RBE) in 8‐38 fractions was performed for 10 patients. The mean interval duration from PT to surgery was 30.1 (range: 4.4‐101.5) months. Preoperative liver function was suitably preserved at hepatectomy in all patients; 14 patients had a Child‐Pugh score of 5, and one patient had a score of 6. The mean total bilirubin, prothrombin, and albumin levels and the indocyanine green retention rate at 15 minutes were 0.85 (range: 0.4‐1.4) mg/dL, 94.2% (range: 77%‐117%), 4.1 (range: 3.4‐4.5) g/L, and 10.2% (range: 5.9%‐18.7%), respectively. The mean maximum tumor size and tumor number before salvage hepatectomy were 3.4 (range: 1.0‐7.2) cm and 2.0 (range: 1‐5), respectively. All recurrent tumors were within the irradiated area and were surgically resected by salvage therapy.

### 3.2 Outcome of salvage hepatectomy

The surgical outcomes of salvage hepatectomy are summarized in Table 2. Major hepatectomy (more than two sections) was performed in seven patients. The mean operation time and blood loss were 396 (range: 143‐654) minutes and 402 (range: 66‐1000) mL, respectively. No postoperative death was recorded.

**TABLE 2:** Outcome of salvage hepatectomy

	Salvage hepatectomy (n = 15)
Major/ Minor hepatectomy	7/8
Operation time (min)	396 (143‐654)
Blood loss (mL)	402 (66‐1000)
Blood transfusion	2 (13%)
Surgical complication	
All	6 (40%)
Clavien‐Dindo ≥ grade IIIa	4 (27%)
Bile leakage	2 (13%)
Pleural effusion	1 (7%)
Skin fistula	1 (7%)
Mortality	0
Postoperative hospital stay (days)	26 (8‐126)

Surgical complications occurred in six cases (40%). Clavien‐Dindo grade IIIa or higher was observed in four cases (27%), consisting of two HCC and two metastatic liver tumor cases: two, one, and one cases of bile leakage, pleural effusion, and refractory skin fistula, required abdominal wall reconstruction using rectal muscle flap. The mean postoperative hospitalization duration of these four patients with Clavien‐Dindo grade IIIa or higher was 59 (range: 13‐126) days; two patients with refractory bile leakage, after partial resection, required an extended hospital stay of 126 and 82 days.

Comparing cases of HCC with those of metastatic liver tumor, no significant differences were observed in the rate of major hepatectomy (38% vs 57%, *P* = .45), morbidity rate (38% vs 43%, *P* = .83), and Clavien‐Dindo grade IIIa or higher complication rate (25% vs 28%, *P* = .89).

### 3.3 Prognosis after salvage hepatectomy

**FIGURE 1:**
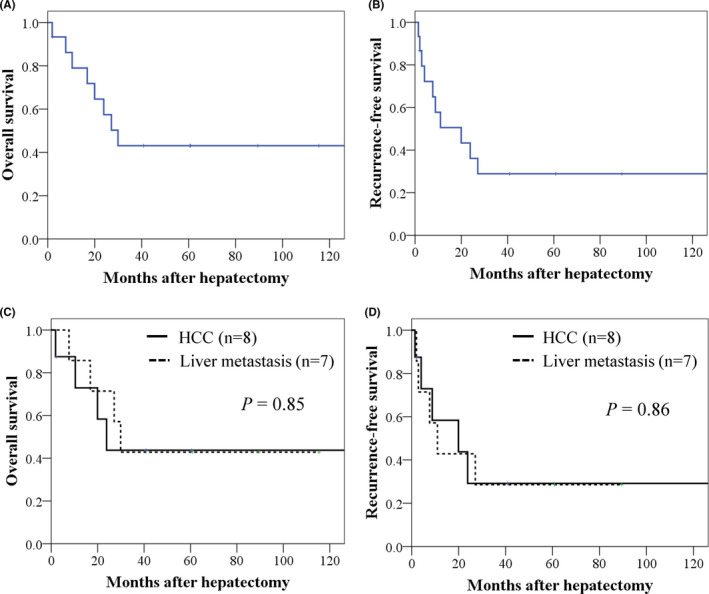
Survival outcomes after salvage hepatectomy. Overall survival (A) and recurrence‐free survival (B) in all patients. Overall survival (C) and recurrence‐free survival (D) in comparison with hepatocellular carcinoma and liver metastasis

The prognosis after salvage hepatectomy is presented in Figure 1. The median OS and RFS of all patients in this study were 29.9 and 19.9 months, respectively; the 5‐year OS and RFS rates were 43.3% and 30%, respectively (Figure 1A,B). In patients with HCC, the median OS and RFS were 23.9 and 19.9 months, and in patients with liver metastasis, the median OS and RFS were 29.9 and 11.0 months, respectively. There were no significant differences in the OS and RFS between HCC and liver metastasis (*P* = .85 and .86; Figure 1C,D).

### 3.4 Macroscopic and microscopic findings

**FIGURE 2:**
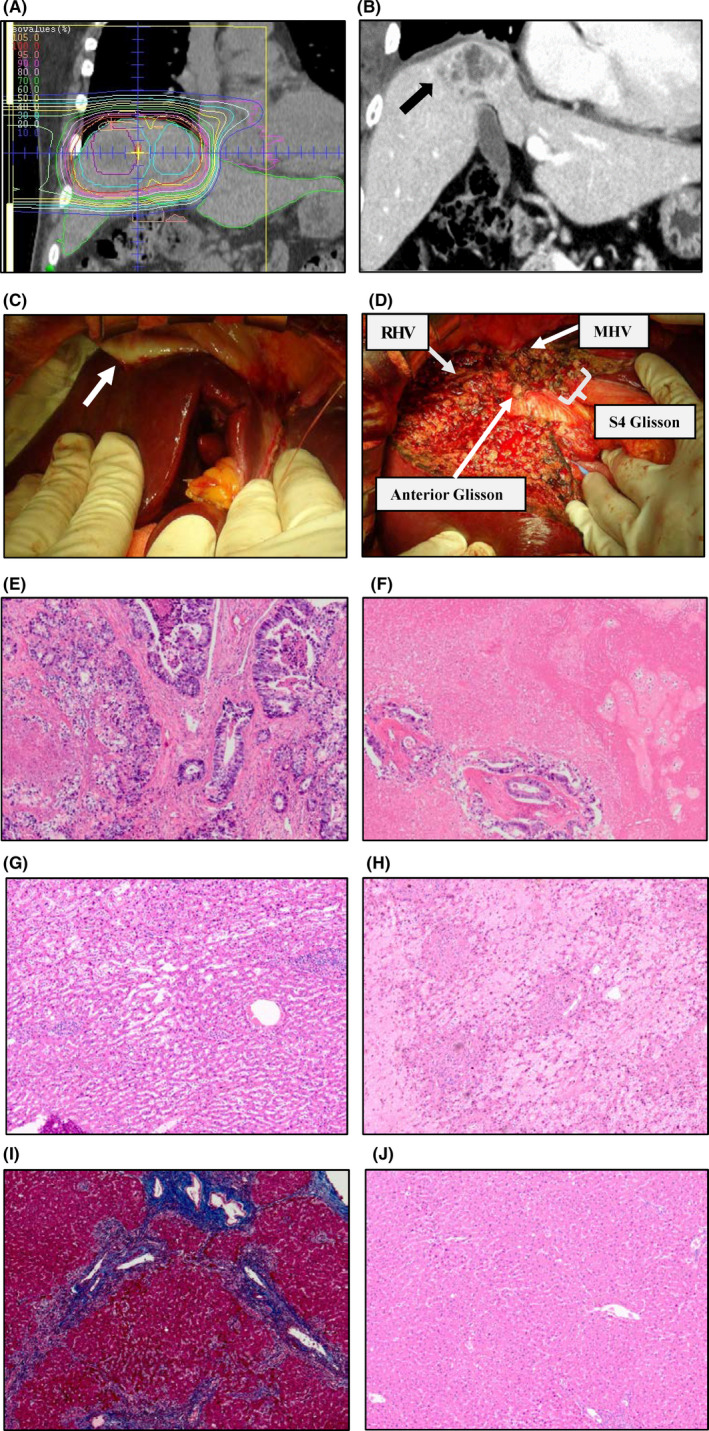
Typical case of salvage hepatectomy (case No. 7). (A) Dose distribution in radiation planning for segment 8 lesion. (B) Computed tomography image at local recurrence (arrow head). (C) Strong adhesion between liver and diaphragm (arrow head). (D) Liver surface after parenchymal dissection. (E‐J) Histological findings. (E) Recurrent tumor, HE ×40. (F) Necrotic tissue around tumor, HE ×40. (G‐I) Irradiated non‐cancerous liver tissue. (G) Sinusoidal dilatation, HE ×40. (H) Loss of hepatocyte, HE ×40. (I) Fibrosis around Glisson, Masson ×40. (J) Non‐irradiated liver tissue, HE ×40. Abbreviations: HE, Hematoxylin and eosin. MHV, middle hepatic vein. RHV, right hepatic vein

A typical case of salvage hepatectomy is presented with macroscopic and microscopic findings in Figure 2. A 66‐year‐old male patient initially underwent PT with proton beam for liver metastasis from rectal cancer (Case 7 in Table 2). The radiation intensity was 66 Gy (RBE) in 10 fractions (Figure 2A). Following 24 months of PT, tumor regrowth was detected on computed tomography (Figure 2B), and the patient was referred to our hospital. Intraoperative findings revealed a strong adhesion between the liver and the diaphragm (Figure 2C); consequently, central bisegmentectomy was performed (Figure 2D).

In recurrent tumors, moderately differentiated tubular adenocarcinoma (Figure 2E) along with the surrounding necrotic tissue (Figure 2F) were microscopically observed. Irradiated non‐cancerous liver tissue displayed unique pathological changes, such as sinusoidal dilatation (Figure 2G), loss of hepatocyte (Figure 2H), and fibrosis of the portal region (Figure 2I). In contrast, the non‐irradiated liver tissue in the lateral segment displayed normal findings (Figure 2J). These pathological changes were observed in the majority of the cases.

### 3.5 Refractory complications

**FIGURE 3:**
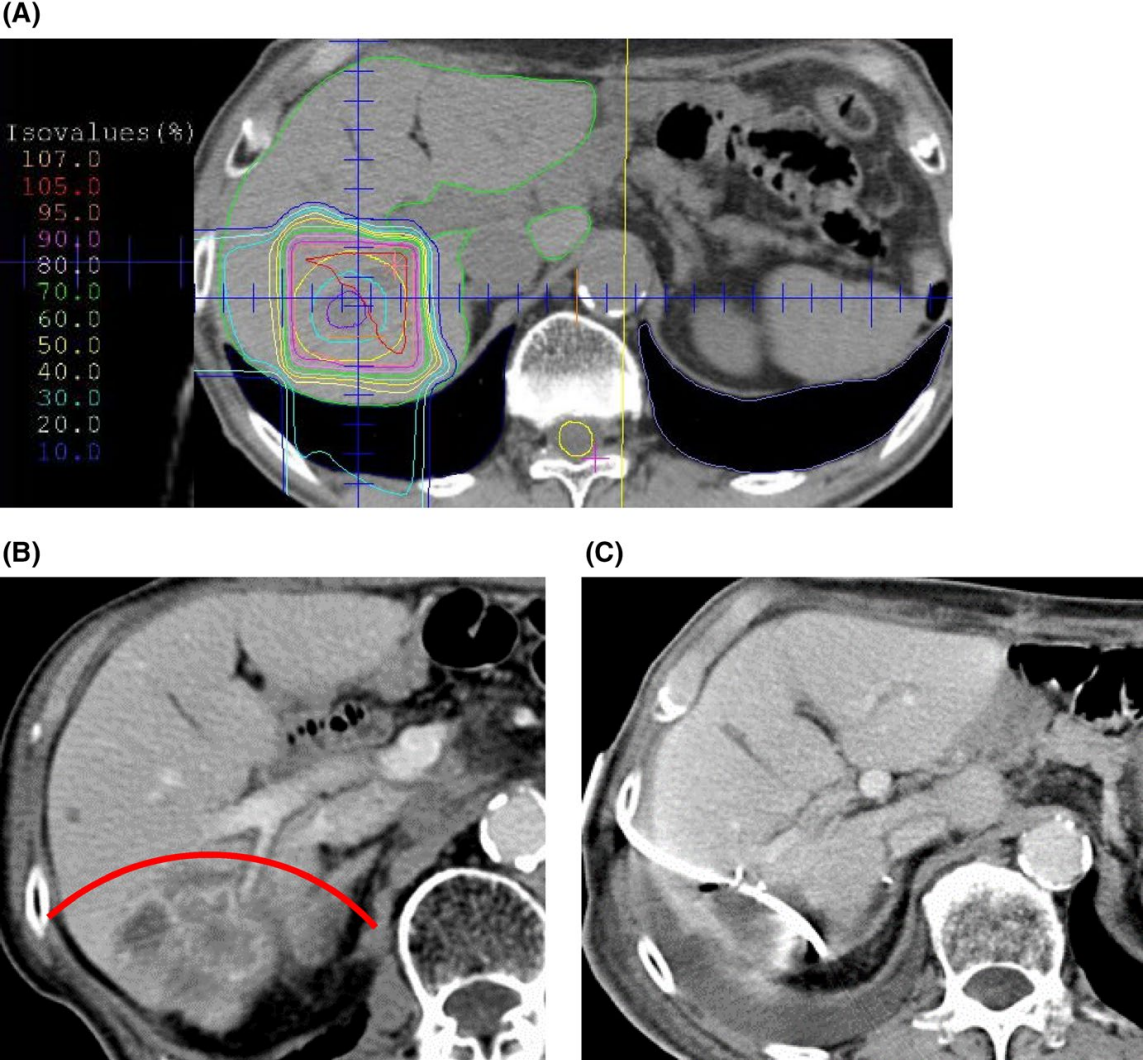
The case of refractory bile leakage (case No. 4). (A) Dose distribution in radiation planning for segment 6 lesion. (B) Computed tomography image at local recurrence. Red line indicates parenchymal resection line performed. (C) Drainage tube placement for postoperative bile leakage

We observed two cases with refractory complications, requiring elongated hospital stay. One case displayed bile leakage (Case 4 in Table 2). A 77‐year‐old male patient was diagnosed with solitary liver metastasis at segment 6, following 4 years of pancreatoduodenectomy for pancreatic head cancer. The patient opted to undergo PT with carbon ion beams with 60 Gy (RBE) in eight fractions (Figure 3A). After 7 months, tumor regrowth was detected by computed tomography (Figure 3B); he was subsequently referred to our hospital. After the evaluation of the tumor status and liver function, we determined that the regrowth tumor was resectable; thus, partial resection in segment 6 was performed. Postoperatively, refractory bile leakage, subsequent local infection, and intra‐abdominal abscess formation were observed; drainage tube placement was required for a prolonged period (Figure 3C). Finally, the patient was discharged on postoperative day 126. A retrospective review of the dose distribution in radiation planning indicated the resection line of the liver parenchyma overlapped with the 80% dose irradiated area (Figure 3A,B).

**FIGURE 4:**
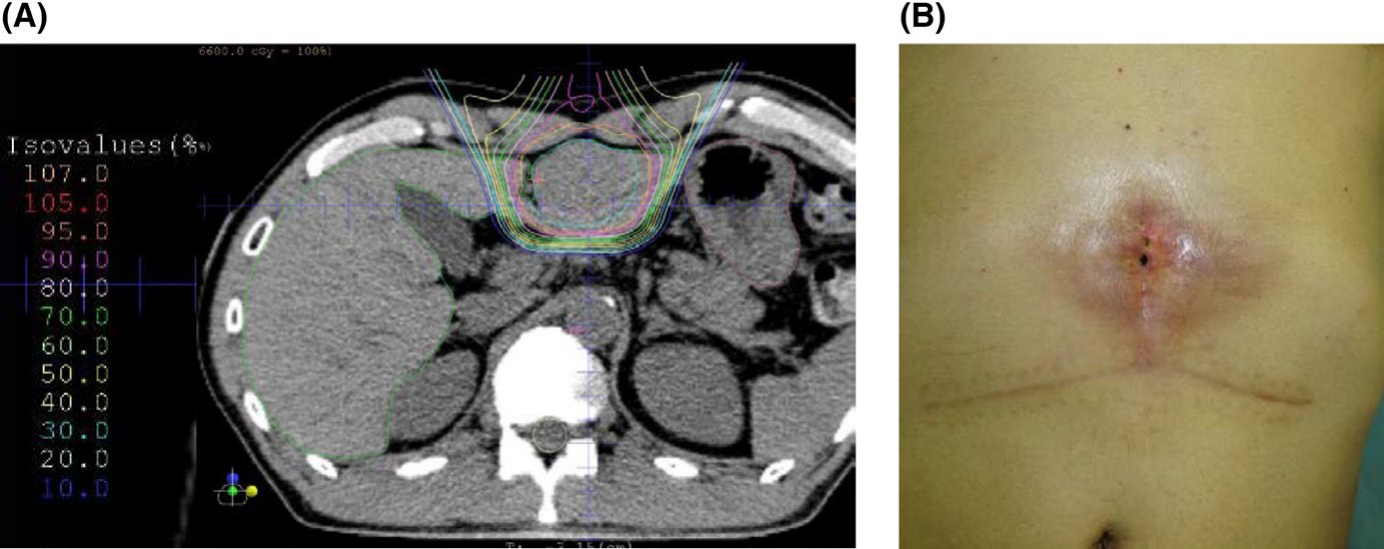
The case of refractory skin fistula after salvage hepatectomy (Case No. 14). (A) Dose distribution in radiation planning shows 90% of maximum dose of proton beam was irradiated to skin and muscle. (B) Postoperative refractory skin fistula and skin discoloration at highly irradiated area

The other postoperative complication observed was refractory skin fistula (Case 14 in Table 2). A 54‐year‐old male patient was diagnosed with HCC associated with chronic hepatitis B. The tumor was solitary, 3.6 cm in size, located in segment 3, and was resectable; however, as per the patient's request, PT with 66 Gy (RBE) of proton beam in 10 fractions was performed (Figure 4A). After 10 months, local recurrence was detected; then, salvage left hepatectomy was performed with a reverse T‐shaped skin incision. Following surgery, wound dehiscence and skin fistula occurred on the midline incision, and it was refractory (Figure 4B). Finally, the patient underwent abdominal wall reconstruction with a rectal muscle flap at 4 months after salvage hepatectomy. The retrospective review of the dose distribution indicated that a 90% dose was irradiated to the skin and the muscle on the incision line (Figure 4A); skin discoloration by irradiation was observed (Figure 4B).

### 3.6 Association between irradiation dose of resection line and refractory complication

We investigated dose distribution in radiation planning in all cases; the association of the effect of increased irradiation with the incision line of the liver or the skin with refractory complications was analyzed. When the cutoff value was set to a maximum dose of 70%, an increased amount was significantly associated with Clavien‐Dindo grade IIIa or higher complications; four (66%) out of six patients and none out of nine patients who received >70% and <70%, respectively, presented grade IIIa complications or higher (*P* = .01).

## 4 DISCUSSION

In the present study, we reported the outcomes of salvage hepatectomy following PT for liver cancer with a certain number of cases. Salvage hepatectomy appears to be feasible; however, there are specific aspects to be considered for surgical indications and procedures to prevent refractory complications.

After PT, surgical resection becomes more challenging because inflammatory changes occur at irradiated areas. As shown in Figure 2C, proton beam irradiation for liver resulted in a strong adhesion in the diaphragm and inflammatory changes around the middle hepatic vein and the inferior vena cava. Therefore, precise handling of the detachment of the liver parenchyma from the diaphragm and the major vessels was required. In this study, major hepatectomy was performed in about half of the cases; consequently, the operation time and blood loss were acceptable.

We experienced a relatively elevated rate of Clavien‐Dindo grade IIIa complications or higher: two bile leakages, one skin fistula, and one pleural effusion. Bile leakage cases were both of partial resection, in which the parenchymal dissection line was on a high‐dose irradiated area. The resultant skin fistula was a case of an open left hepatectomy with a midline incision of the skin; a high‐dose proton beam irradiation was performed. In this case, the irradiated skin was discolored at the time of hepatectomy. As a result of refractory complications, it was speculated that wound healing did not occur effectively due to an excessive dose of particle beam irradiation. In this study, the cutting of the liver or skin in a high‐dose irradiated area was significantly associated with severe complications. To prevent complications, parenchymal dissection and skin incision should be made distant to high‐dose irradiated areas. In this regard, major hepatectomy may be a preferable alternative for adequate liver function to avoid dissection of high‐dose irradiated liver. In fact, no bile leakage was observed in all cases of major hepatectomy. Regarding skin incision, the laparoscopic approach may be effective to avoid highly irradiated skin incision, especially for left‐side tumors because open hepatectomy generally requires a midline skin incision. Another concern was the safe radiation dose of the particle beam for salvage hepatectomy. In the present study, 70% of the maximum dose is significantly associated with severe complications. However, there were no sufficient data regarding the safe radiation dose for surgical dissection. Therefore, further investigation is warranted to explore this issue.

There were few reports about the pathological changes in the liver following PT, whereas conventional radiotherapy was outlined.^11^ In this study, the irradiated non‐cancerous liver tissue surrounding the tumor displayed sinusoidal dilatation, loss of hepatocyte, and fibrosis in the majority of the cases. As for the pathogenesis of liver damage after irradiation, a non‐human primate model revealed conventional radiation‐induced veno‐occlusive changes, resulting in sinusoidal congestion and fibrosis.^12^ Interestingly, in the present study, liver function was well‐preserved after PT in the majority of the patients; hence, it was deemed feasible for major salvage hepatectomy in half of the cases. This observation may depend on the high‐dose concentration of PT for liver tumor. Normal pathological findings in the non‐irradiated liver tissue outside the irradiated area provided suitable evidence (Figure 2J).

With regard to prognosis, the 5‐year OS and RFS rates in all cases were 43.3% and 30%, respectively; there was no significant difference between HCC and liver metastasis. The 5‐year OS rate of salvage hepatectomy for recurrent HCC following locoregional therapy, such as radiofrequency ablation and/or transarterial chemoembolization, was 38%‐69%.^13^, ^14^ Although the number of patients in the present study was limited, we found that salvage hepatectomy is a potential rescue therapy to prolong survival following local recurrence of HCC and metastatic liver tumor after PT.

According to the guidelines of the American Association for the Study of Liver Diseases and the Japan Society of Hepatology, the methods for HCC treatment with curative intent include surgical resection, radiofrequency ablation, and liver transplantation.^15^, ^16^ Radiation therapy, including PT, has not been standardized in these therapeutic algorithms yet. However, a positive local control rate for HCC has been reported.^3^, ^4^, ^5^, ^6^ This includes advanced cases with portal venous^17^ or inferior vena cava tumor thrombus.^4^ Moreover, a non‐randomized controlled study comparing proton beam therapy to surgical resection for resectable HCC is ongoing in Japan.^18^ For colorectal liver metastasis, surgical resection remains the standard treatment^19^; however, the local control rate of particle therapy for liver metastasis has been reported to be 61%‐66%,^20^, ^21^ and several studies displayed an improved local control rate by dose escalation manner with carbon ion and proton beam.^22^, ^23^ Additionally, some patients prefer receiving PT as a less‐invasive therapy, even for resectable tumors. Considering these situations and the results of the present study, primary care physicians and hepatologists should recognize and explain to patients that surgery remains the first choice for resectable tumor and that if local recurrence following PT occurs, treatment selection and salvage hepatectomy become more difficult.

The limitations of the present study were its retrospective nature and the small sample size. Currently, the number of salvage hepatectomies conducted after PT is limited and only a few case reports are referable.^8^, ^9^ In 14 years, salvage hepatectomy was performed in only 17 out of 110 patients who were treated with PT in Hyogo Ion Beam Center and experienced local recurrence. There are some reasons for this small number: difficult pattern of local recurrence, liver dysfunction after PT, and less knowledge concerning salvage hepatectomy. However, the number of patients undergoing PT for liver cancer has increased in the last two decades^3^ because of improved techniques, higher numbers of facilities, and expanded use for resectable cases and metastatic liver tumor. These circumstances add weight to salvage hepatectomy in the treatment of recurrent liver tumor. The outcomes obtained from this study can be a guide for surgeons to perform proper treatment selection and perioperative management of local recurrence after PT. We believe that this study may contribute to spreading the knowledge about salvage hepatectomy.

In conclusion, salvage hepatectomy for local recurrence after PT is feasible and effective; however, it generates an increased risk of refractory complications associated with previous irradiation. Therefore, the first‐line therapy for resectable liver tumor should be carefully determined, considering the possibility and risk of second‐line therapy.

## DISCLOSURE

**Funding:** This research did not receive any specific grant from funding agencies in the public, commercial, or not‐for‐profit sectors.

**Conflict of Interest:** Authors declare no conflict of interests for this article.
